# Analyzing the impact of deep learning algorithms and fuzzy logic approach for remote English translation

**DOI:** 10.1038/s41598-024-64831-w

**Published:** 2024-06-24

**Authors:** Xiuying Han

**Affiliations:** Shanghai Normal University Tianhua College, Shanghai, 201815 China

**Keywords:** Deep learning, English translation, Fuzzy algorithm, Precision improvement, Engineering, Mathematics and computing

## Abstract

A remote English translation is used for assisting with on-demand support for adaptable sentence conversion and language understanding. The problem with on-demand translations is the precision verification of the words used. This article addresses the precision problem by assimilating deep learning and fuzzy decision algorithm for remote translation support. The method named Fusion-dependent Precision Translation Approach (FPTA) conducts a series of recurrent validations on word usage and sentence completion for the given inputs. First, the completed sentences are verified using the understandability and meaning intended using deep learning in two recurrent layers. The first layer is responsible for identifying word placement and understandability and the second is responsible for meaning verification. The recurrent training is tuned using a fuzzy decision algorithm by selecting the maximum best-afford solution. The constraint’s understandability and meaning are augmented for tuning the outputs by preventing errors consequently. In precise, the error sequences are identified from the first layer for fuzzification across various inputs. This process improves the word adaptability from different languages reducing errors (12.49%) and improves the understandability (11.57%) for various translated sentences.

## Introduction

A remote English translation is a technology that is used anywhere or any place to understand the content. Remote English translation technology is used to translate the content from one language to another language^1^. The precision issues in remote English translation are critical, needing robust solutions for better accuracy and reliability. Poor translation quality results from the inability of current approaches to handle the complexities of on-demand and low-resource translations. There are many obstacles to precision because of the complexity of language, such as the wide variety of sentence forms and idiomatic idioms. To account for the complexity of the original language, this work presents a fusion-dependent quality translation method that employs deep learning and fuzzy logic^2^. This approach improves precision by saving time during verification, decreasing the possibility of errors, and making the most of specific word choices. Automated English translation services use AI-based systematic approaches. Software that is highly effective could be designed to understand language or translate texts automatically using artificial intelligence. An AI-powered system gives precise interpretations of the data and supplies relevant details via translation services. There is trust among users that the translations generated by the AI platform are accurate^3^. The systems become more practical with an AI-powered method, which improves the translation process. The technology uses DL algorithms to remotely translate from English to another language^4^. Utilizing the DL algorithm with the sign language interpretation and communication system has several advantages, one of which is the simplification of comprehension^5^. Device mobility is enhanced by the DL algorithm’s creation of an exact English translation. Turning on the DL algorithm’s real-time translating feature is entirely up to the user^6^.

The use of fuzzy approaches is common when dealing with programmatic issues. Decisions are often better made with the help of fuzzy strategies^7^. In order to make English translation systems more efficient, fuzzy approaches are used. The use of fuzzy algorithms in translation systems is a common practice for providing customers with high-quality English translators^8^. Fuzzy algorithms’ primary strength lies in their feature extraction capabilities. The collected variables and attributes can be used to generate data that is acceptable for English translation^9^. The English translation systems are another domain were fuzzy logic-based paradigm is employed. To determine if translation is necessary, the fuzzy logic approach is often used^10^. make sure that your criteria are tailored to produce the most reliable data for fuzzy match. The fuzzy matching strategy saves researchers time and energy^11^. English-language data translation accuracy is fuzzy matching’s main goal. Fuzzy matching can boost ESL translation system performance and benefits^12^. FPTAs employ deep learning and fuzzy logic to grasp complex input languages. Start by creating a translation system that compares data from multiple languages. Further study fixes the input phrase’s meaning to improve the model’s prediction and output complexity. Because FPTA accepts ambiguity, communication is clear.

The metrics that matter the most, including word usage, verification time, blunders, and understandability, have all shown significant improvement. Together, fuzzy algorithms and a two-tiered deep learning approach allow the system to improve context-aware translation accuracy while decreasing verification time. When looking for specific process data, machine learning (ML) techniques and methodologies are often applied. Algorithm performance is enhanced by ML approach, which boosts detection accuracy^13^. Software that can translate among English and various other languages sometimes makes use of ML-based approaches. Systematic translations make use of deep learning (DL) classification algorithms. This is because the DL classification model improves translation accuracy by integrating language expertise with model features^14^. The DL categorization paradigm facilitates language translation systems. Reverse translation strategies based on hierarchical transfer learning are employed by machine translation systems for English-to-language translation^15^. Using the transfer learning method effectively here entails primarily comprehending the initial purpose of the content. An attention mechanism provides an appropriate optimization procedure as part of a translation approach^16^. Automatic English-to-Spanish translation systems benefit from the transfer learning technique. Standard practice dictates that English translation systems employ a neural machine translating (NMT) model for optimal results^14,16^. Research after research has proven that ETSs frequently employ NMT. Effective application of deep learning techniques, neural model layers, hidden operations, and neural parameters improves the translation system’s efficiency. Using an intelligent recognition model, the NMT model finds translationally important elements. Improved recognition quality helps translation systems in the long run^17^.

This paper introduces a novel strategy for addressing the difficulties of precise remote translation by combining deep learning and fuzzy logic. It streamlines the process, cuts down on verification time, and improves on-demand translation efficiency by employing a two-layered deep-learning method for translation verification. Overall, the study proposed a wide range of evaluation criteria for translation quality and efficiency, including usability, mistakes, word utilization, verification time, and word adaptation. The study highlights the value of FPTA by zeroing in on the challenge of accuracy verification in fast translation. The importance of FPTA in satisfying user demands is brought to light by problems with real-time translation. Take a look at the submissions below:

- Enhancing the understandability of phrases translated from foreign languages to English through the use of a deep learning and fuzzy fused translation technique.
- Conducting separate analysis and classification to validate meaning and reduce understandability constraints for various mistake sequences.
- Analyzing the fuzzy fused learning process intensively using data to confirm the effectiveness of the suggested method.

## Related works

Yang et al.^18^ suggested a method for Neural Machine Translation (NMT) that relies on consensus at the sentence level. Applying the proposed method allows for the objective training of NMT systems. The sentence-level agreement method is the main tool for retrieving precise semantic data at the sentence layer. The agreement approach improves the efficiency of offering services to users. The effective range of NMT systems is increased by the proposed method.

Zhang^19^ introduced a deep learning (DL) model-based automated method for NMT fault detection. To find grammatical mistakes in a sentence, the technique use a deep producing model. The error-correct function is used to detect grammatical errors. In order to decrease calculation delay, the newly presented detection approach employs DL methods. An improvement in the precision of the error detection procedure is demonstrated experimentally by the new approach.

For quantum natural language processing (Q-NLP) in NMT systems, Abbaszade et al.^20^ developed a novel compositional vector-based semantics. Among the many services provided to customers by the developed Q-NLP is an algorithm known as long short-term memory (LSTM). In machine translation, the quantum circuits of a phrase are examined. For NMT systems to construct a sentence, the best semantics are supplied. The developed Q-NLP enhances the systems’ performance and makes them more feasible.

Using a recurrent context model, Zhao and Liu^21^ created a document-level approach to NMT. The method’s primary objective is to encode the extensive information conveyed in a sentence. In this case, the key function of the context model is to translate the sentence’s exact substance. Service delivery speed is enhanced by the context model, which enhances the sentence level sustainability ratio. An increase in the usable performance spectrum of NMT systems is achieved by the created technique.

A method for multimodal neural machine translation (MNMT) was suggested by Zhao et al.^22^ and is called word-region alignment (WRA). We examine the real semantic relationship for MNMT between visual and textual modalities. In order to translate the content for the systems, the suggested WRA method employs a recurrent neural network (RNN). Features important to translation can be found using the WRA method. As compared to previous methods, the suggested WRA methodology improves translation accuracy.

A model for machine translation (MT) from French to English was proposed by Tian et al.^23^ using a transmission network. The datasets are trained using significant keywords by the suggested model. Specifically, the model employs an attention mechanism to spot content flaws. A user-friendly translation procedure is created by the attention mechanism. The suggested model improves MT system performance by making data translation more accurate.

A translation method was created by Shen and Qin^24^ using a new approach for deep neural networks (DNNs). In order to construct an accurate translation model, the created model extracts the Source language sentence extraction (SLSE). Here, we employ the SLSE approach to generate data that enhances the content. In order to speed up the data translation process, the data mining technique is employed. Machine translation systems are made more efficient with the proposed model’s increased translation accuracy.

Translation systems were given a new tool by Li et al.^25^: the transformer fast gradient technique (FGM) using relative positional embedding (TF-RPE). In this case, the language data needed for translation is extracted using a positional encoding. The FGM offers a training technique that effectively decreases the energy consumption ratio during translation. When it comes to system reliability, the FGM approach is tops. The effective range of translation systems is enhanced by the proposed FGM approach.

Statistical machine translation (SMT) was developed by Zhang et al.^26^ with the help of deep learning (DL) algorithms. The method relies on a feature extraction methodology to glean the crucial features. When used in data translation, the extracted feature yields the best possible results. In this case, we employ a secure classifier to categorize the statistical variables that improve translation system efficiency. Users can get translations of superior quality thanks to the developed SMT approach.

To aid those who are deaf or hard of hearing, Rajalakshmi et al.^27^ suggested a hybrid deep neural network that relies on vision to recognize sign language. The architecture employs a number of feature extraction techniques, including those using spatial, temporal, sequential, abstract, and discriminative features. The results show that it is superior when tested on a dataset of Indo-Russian sign language with several signers. More research is required before generalization and implementation in the real world are possible.

A data augmentation strategy for English-Vietnamese NMT systems was proposed by Pham and Pham^28^. Synthetic parallel data translation is the primary application of the suggested method. Grammar mistakes are identified, which gives the data needed for the error fixing procedure. The suggested approach gathers content and monolingual text for translation, which speeds up the identification process. Users are provided with an effective data translation system by the proposed method.

A sentence embedding strategy for NMT systems was introduced by Unanue et al.^29^. Specifically, NMT makes use of the presented method as an automated translation method. The data is trained to construct sentences by identifying sequences of words and attributes. Translation systems are made more efficient by including low-resource content in their output. According to the experimental findings, the performance range of the systems is increased by the introduced technique.

Rajalakshmi et al.^30^ presented a system for sign language recognition that uses a Hybrid Neural Network Architecture to understand the language spoken by the deaf and hard of hearing. Using a 3D Convolutional Net, it uses semantic spatial multi-cue feature detection for hand and face detection. Achieving 99.76% accuracy for static Isolated Sign Recognition and 99.85% accuracy for dynamic Isolated Sign Recognition, the system utilized a new dataset for Russian and Indian Sign Language. To determine the model’s usefulness and performance in practical situations, additional research is needed.

The NMT model put forth by Li et al.^31^ is based on latent feature encoders (LFEs). The model incorporates feedback to generate translation-relevant delay features. By encoding latent characteristics using LFE, we can reduce computation-related energy usage. Improved translation accuracy is a direct result of the useful service that latent characteristics give to users. The suggested NMT model enhances the systems’ performance range and overall quality of service (QoS).

In order to control the training and use photorealistic, high-quality films for sign language understanding, Natarajan and Elakkiya^32^ suggested a generative adversarial network. Using human images and skeletal position data as input, the proposed method produces high-quality films. While in the generator phase, the proposed model uses a network such as U-Net to construct target frames from skeleton positions. Metrics that were considered had an average inception score of 8.72, a structural similarity score of 0.92, and a peak signal-to-noise ratio of 28.7. The first table provides a synopsis of the relevant literature (Table 1).

**Table 1:** Comparison Analysis of Related Works.

Author and Year	Methods	Objective	Findings
Yang et al.^18^, 2022	Neural Machine Translation (NMT)	Increases the efficiency level in providing services to users	Improves the effective range of NMT systems
Zhang^19^, 2023	Deep learning (DL)	To detect grammatical errors in a sentence	Increases the accuracy of the error detection process
Abbaszade et al.^20^ , 2021	Quantum natural language processing (Q-NLP)	Aiming to develop the automatic language processing system	Designed Q-NLP improves the feasibility and performance level of the systems
Zhao and Liu^21^, 2023	Recurrent context model	Aim of the method is to encode the large content which is presented in a sentence	Significantly improves the performance range of NMT systems
Zhao et al.^22^, 2021	Word-region alignment (WRA) approach	Actual semantic correlation between visual and textual modalities for MNMT is analyzed	WRA approach increases the accuracy of the translation process
Tian et al.^23^, 2022	Transfer network-based French-to-English machine translation (MT)	To identify the defects which are presented in a content	Increases the accuracy of data translation which enhances the effectiveness of MT systems
Shen and Qin^24^, 2021	A new deep neural network (DNN)	To reduce the time of processing for translating the data	Increases the translation accuracy that improves the efficiency of machine translation systems
Li et al.^25^, 2022	Fast gradient method (FGM)	To extract the linguistic information for the translation process	Maximizes the reliability level of the systems
Zhang et al.^26^, 2022	Statistical machine translation (SMT) using deep learning (DL) algorithms	To create the automatic translation process	Provides high-quality translation content for the users
Rajalakshmi et al.^27^, 2023	Vision-based hybrid deep neural network	Identification to help the speech and hearing handicapped	Generalization and deployment in the actual world necessitate more study
Pham and Pham ^28^, 023	Data augmentation method for English-Vietnamese NMT systems	Creating synthetic parallel data translation process	Provides an effective data translation system for the users
Unanue et al.^29^, 2022	Sentence embedding technique	Creating the automated translation technique in NMT	Increases the performance range of the systems
Rajalakshmi et al.^30^, 2022	Hybrid Neural Network Architecture	To develop the sign language recognition systems	Ensures the high detection accuracy
Li et al.^31^, 2021	Latent feature encoder (LFE)	Produces relevant latency features for the translation process	Model improves the overall quality of service (QoS) and performance range of the systems
Natarajan and Elakkiya^32^	Generative adversarial network	To regulate the training and photo realistic high-quality videos for understanding sign language	28.7 peak signal to noise ratio value, 0.92 structural similarity score, 8.72 average inception score

With on-demand support for adaptable sentence conversion, the translation system can dynamically adapt its translations based on context and specific requirements. This elucidates that the operation takes into account the nuances of the given other language texts and provides a precise and accurate translation in the English language. Moreover, these English translations aid in language understanding by helping users comprehend content written or spoken in the English language. Many significant areas of research are in agreement with the strategy, including a sentence-level agreement method for Neural Machine Translation (NMT), an error detection technique for NMT systems using deep learning models, a document-level way for NMT using a recurrent context model, a suggested word-region alignment approach for multimodal neural machine translation, a translation technique based on a new deep neural network algorithm, and a latent feature encoder (LFE)-based NMT model. These researches highlight the difficulties of remote English translation and the unique and all-encompassing answer that combines deep learning and fuzzy logic in translation.The development of FPTA has mostly focused on improving the translating model and making translations more accurate. To improve the accuracy of remote translation support, the Fusion-dependent Precision Translation Approach (FPTA) employ deep learning and fuzzy decision theory. With regular tests on syntax and phrase completeness, FPTA guarantees reliable translations. The methods NMT-LFE^29^, SLSE-DNN^24^, and MNMT^22^ are utilized to evaluate the efficacy of the Fusion-dependent Precision Translation Approach. Current methods are supplemented with Neural Machine Translation (NMT) to ascertain the language. Most of the existing methods for language recognition make effective use of NMT.Hence, this study compared NMT-LFE^29^, SLSE-DNN^24^, and MNMT^22^.

## Fusion-dependent precision translation approach

Online translation services that may be accessed whenever needed greatly improve the quality of cross-lingual communication and understanding. We can provide more versatile sentence conversion options and enhance real-time language comprehension with these translations. Among the primary objectives of distant English translation are the dissemination of information, the improvement of cross-cultural communication, and the expansion of access to worldwide materials. Modern society relies on this technology to immerse itself in many cultures, communicate internationally, and acquire new languages. In the information environment, this serves to foster inclusiveness and global involvement. In today’s interconnected world, it plays a crucial role in connecting individuals and businesses across borders and languages. One of deep learning’s numerous uses is better word and phrase translation. Because of this, they can translate accurately and meaningfully. Translation coherence and mistakes are checked by the completion layer. Word order, tense, and alignment must be carefully considered to achieve good grammar in translations. The meaning layer must ensure the translated information matches the original. Deep learning machine translation improves word and phrase accuracy and context relevance. Deep learning data allows for the most efficient and cost-effective translations. This can be done with fuzzy logic. Fuzzy finds potential translations using metrics for similarity and other language aspects that match the goal meaning. This method lets customers choose full, precise translations. Using fuzzy logic combined deep learning enables for more student-specific training.

The approach makes use of the student profiles, pertinent data, performance history, and musical scores. The use of fuzzy control systems to enhance decision making is explored in this pertinent knowledge. As linguistic variables, the fuzzy method investigates student performance in relation to tempo knowledge, pitch accuracy, and musical skill. The student’s musical expertise is encoded using variables and the fuzzy rule. The rules help the machine make decisions by establishing links between language factors. As a first step, fuzzification converts the raw data into fuzzy values using specified membership functions so that fuzzy inference may be used. The core of the system, fuzzy inference, interprets the fuzzified data according to the defined rules and then makes suggestions or conclusions using fuzzy language. For example, based on a student’s performance, we can modify the difficulty level or propose certain musical exercises; these suggestions are defuzzified to give real, actionable guidance. With a methodical approach that flawlessly combines deep learning with fuzzy logic, the Fusion-dependent Precision Translation Approach (FPTA) is designed to solve precision concerns in distant English translation.

Following are the main components of the FPTA process:

- (i) Using deep learning algorithms for training translation models is the first step in doing FPTA’s startup phase. utilizing a large quantity of matched bilingual data is an initial stage in training a model utilizing deep learning techniques. Once the model has received this basic training, it can start to see patterns and find correlations between the source and target English languages.
- (ii) Two-Pronged Deep Learning Strategy for Accurate Translation: The FPTA system uses two-stage deep learning to improve translation accuracy. A large dataset is needed to train the translation model to recognize complicated linguistic patterns. An accurate translator must recognize idioms and other language patterns.
- (iii) Check that the Prominence Layer decodes data as intended. This extra step ensures that all translated models match the original documentation, raising translation quality.
- (iv)The FPTA training method shows fuzzy logic’s capacity to manage language ambiguities and imprecisions. Fuzzy logic handles words and sentences with several meanings, making language comprehension easier.
- (v) Fault-tolerant deep learning models in FPTA must adapt to new data and modifications. Regular data updates increase these models’ translating ability. Plasticity allows the translation to improve accuracy and ease over time.
- (vi) Evaluation measures: FPTA uses metrics designed to measure translation accuracy and impact to assess its performance. This is an entire list of measures: Consider “word adaptability,” “understandability,” “verification time,” “errors,” and “language utilization” in this context. These measurements show the method’s pros and cons.

Finally, the FPTA accomplishes better, more accurate, and relevant translations by training translation models with deep learning pattern identification skills and integrating fuzzy logic to handle linguistic uncertainties. Effective remote translation into English presents unique obstacles; this all-encompassing strategy can help. In a two-stage procedure, the FPTA integrates fuzzy logic via deep learning to deliver translations that are both accurate and relevant to the context of live translation services. By taking linguistic barriers into account, this method guarantees generalizability. For quicker and more accurate translation, it is necessary to concentrate on meaning verification and enhance the model’s training. With the use of fuzzy FPTA, we can make our translations more readable and make less mistakes. Due to its adaptability, translations across cultures tend to be accurate and courteous. Aiming to produce precise and personalized translations, FPTA works within the limitations of current technology. Research based on data on translation quality is used to produce practical, actionable recommendations. So, the study will present data from an external source (https://www.kaggle.com/datasets/devanshusingh/machine-translation-dataset-de-en)later on in the essay. In particular, FPTA benefits from the phrase patterns, linguistic contexts, and nuances provided by these datasets. With the Kaggle dataset, you can train and fine-tune the FPTA model to produce more accurate and trustworthy results. By using it, users are able to observe how the FPTA performs compared to other approaches, handle data of poor quality, and apply its knowledge to new situations. In order for FPTA to work with different languages and contexts, this general idea is crucial. The data is summarized with the description in Table 2.

**Table 2:** Data source description.

Parameter name	Value De-en	Description German to English
Training data	28K	words utilized in the training set
Testing data	1K	Words or non-repeated per sentence utilized in the testing set
Sentences	10.3K	Sentence utilized used for analysis
Words	64.2K	Words utilized used for analysis
Max. word placement/sentence	18	Number of words repeated or non-repeated per sentence

Using deep learning and a fuzzy algorithm, the texts written in other languages are fed into the English translation process. The translation process begins with an examination of the source texts and words in the target language. After the translation procedure, the deep learning technique is used in determining the completion of the translated sentence and then the meaning of the texts in two layers. The deep learning technique is happening with the two recurrent layers to estimate the correct translated texts or phrases. After the deep learning process, the best afford translated texts with precise meaning are identified by using the fuzzy logic algorithm. The recurrent training in the proposed method involves tuning with a fuzzy decision algorithm to select the best-afford solution. The understandability and meaning of constraints are enhanced to prevent errors. Specifically, error sequences are identified from the first layer and subjected to fuzzification across different inputs. This process improves word adaptability, reducing errors and enhancing the accuracy of translations from various languages. The source texts in the other languages serve as inputs to the English language translation process. To guarantee that the representations of outputs are accurate representations of input data, the Fusion-dependent Precision Translation Approach (FPTA) uses repeated validations. An important question comes up about the possibility of using deep learning—more especially by using two recurrent layers—to improve translation target monitoring and early warning signal interpretation. This investigation aims to find out how much FPTA’s accuracy and performance may be improved by incorporating deep learning into its translation quality assurance process. The following describes the steps involved in passing in texts written in foreign languages as input: Eq. (1) given below:

1 $$ X = \left\{ {x_{1} ,x_{2} , \ldots ,x_{n} } \right\} X^{*} = \mathop \sum \limits_{n = 1}^{{}} \left\{ X \right\}\underbrace {{\phantom{0}}}_{{\phantom{0}}}W = \left\{ {w_{1} ,w_{2} , \ldots ,w_{n} } \right\} X^{*} = \mathop \sum \limits_{n = 1}^{{}} \left\{ {W,X} \right\} = \mathop \sum \limits_{n = 1}^{{}} \left\{ {\frac{{\left( {W,X} \right)}}{\left( W \right)}} \right\} = \mathop \sum \limits_{n = 1}^{{}} \left\{ {\frac{{\left( {W,X} \right)\left( W \right)}}{\left( X \right)}} \right\} = \mathop \sum \limits_{n = 1}^{{}} \left( {\left( X \right)\left( W \right)} \right) \} $$

$$ X{ }$$ stands for the texts in the other languages, and $${ }W$$ represents the process of feeding them into the translator.The translation method is happening with the other language text as the input $$ \left( {X^{*} = \mathop \sum \limits_{n = 1}^{{}} \left\{ {W,X} \right\}} \right)$$. Now the translation process takes place with the given input to estimate the perfect meaning and then the completion of the phrase. The English translation process involves converting text from another language into English. It typically accompanies s a sequence of procedures to ensure accurate and meaningful translations. Firstly, the input text in the source language is analyzed, breaking it down into sentences and individual words. This may involve syntactic and semantic analysis, word alignment, and contextual understanding to produce a fluent and contextually appropriate translation in English. Post-processing steps may be applied to refine the translation and ensure linguistic accuracy which is described in Eq. (2)

2 $$ P\left( {w_{i} } \right) = \frac{{P\left( {\frac{{x_{i} }}{{w_{i} }}} \right)}}{W} W_{P} = \frac{w \times i \times m}{W} P_{ij} = \sqrt {\left( {w_{i} - w_{j} } \right)\left( {w_{i} - w_{j} } \right)} P_{ij} = \sqrt {\mathop \sum \limits_{n = 1}^{{}} \left( {x\left( {w_{i} } \right) - x\left( {w_{j} } \right)} \right)^{2} } P = \left\| {P_{11} \cdots P_{1n} \vdots \ddots \vdots P_{n1} \cdots P_{nn} } \right\| \} $$

**Figure 1:**
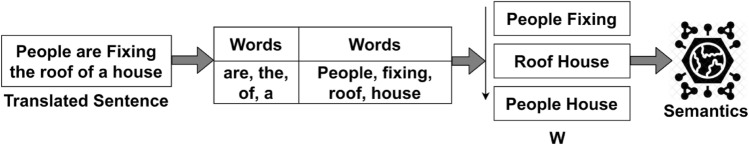
Semantic analysis using sample sentence.

The method of translation with the other language texts as the input is described by the equation as followed given above. Where $P$ is represented as the translation procedure, $i$ is denoted as the analyzing operations of the given input, $j$ is represented as the semantic analysis in the translation procedure. The translation procedure is proceeding by determining the other language texts $\left( W \right)$ and its sending procedure $\left( x \right)$ as the input to the process $\left( {P_{ij} = \sqrt {\mathop \sum \limits_{n = 1}^{{}} \left( {x\left( {w_{i} } \right) - x\left( {w_{j} } \right)} \right)^{2} } } \right)$. After the translation process, the translated texts are sent to the deep learning technique to determine the completion and the meaning of the translated texts by using the two recurrent layers. The semantic analysis using a sample sentence from the given data source is presented in Fig. 1.

The translated sentence is augmented for splitting up words based on common occurrences and complex words. In any translated sentence the $$ W$$ is extracted for semantics operation. The above usage is common for $$ X$$ for all $$ P$$ such that $$ j$$ is uniform regardless of different inputs (Fig. 1). At first, the completion of the translated sentence in layer -1 is evaluated by using the deep learning technique. In the deep learning technique for the translation process, the verification of sentence completion takes place in layer 1 of the model. This layer focuses on ensuring that the translated sentence is grammatically correct and syntactically well-formed in the target language. It examines factors such as word order, verb agreement, noun-pronoun agreement, and other grammatical rules. By verifying completion in this initial layer, the model aims to produce accurate and fluent translations that maintain the structural integrity and grammatical coherence of the translated sentence, enhancing the whole quality of the output of translation. The process of checking the completion of the translated sentence by using the deep learning technique is described by the Eq. (3) as followed:

3 $$ K_{i} = \mathop \sum \limits_{n = 1}^{{}} \left( {x_{i1} ,x_{i2} , \ldots ,x_{in} } \right) K_{i} = \mathop \sum \limits_{n = 1}^{{}} \left( {1 - \sqrt {\mathop \sum \limits_{K = 1}^{{}} \left( {w_{i} \left( {x_{i} } \right) - w_{j} \left( {x_{j} } \right)} \right)} } \right) where j = 1,2, \ldots ,n K = \left[ {K_{1} ,K_{2} , \ldots ,K_{n} } \right] where \mathop \sum \limits_{i = 1}^{{}} K_{i} = 1 K = i \times k = \left[ {k_{i} ,k_{j} , \ldots ,k_{n} } \right] \} $$

where $K$ is represented as the complete estimation of the translated texts or phrases. And in this process, it checks whether the full sentence is completely translated or not. The English words are sent as an addition to the deep learning procedures. It ensures that the entire input sentence is properly processed and converted into the target language. This involves analyzing the sentence structure, identifying the main components, and generating the corresponding translation for each part. Based on the sentence formation the completion analyzing factors discussed above is presented in Table 3.

**Table 3:** Sentence formation analysis.

Words	Structure	Components	Translation	Completion
*10*	Yes	✰✰✰	Before	0.58
			After	0.71
	No	✰✰	Before	0.53
			After	0.65
*12*	Yes	✰✰✰✰	Before	0.74
			After	0.83
	No	✰✰	Before	0.78
			After	0.81
*16*	Yes	✰✰✰✰	Before	0.75
			After	0.86
	No	✰✰	Before	0.55
			After	0.65
*18*	Yes	✰✰✰✰✰	Before	0.81
			After	0.89
	No	✰✰✰✰	Before	0.83
			After	0.87

The completion of a sentence is determined based on the components observed. The sentence $$ i$$ is used for $$ j$$ for $$ K$$ validation using $$ \left( {x_{i1} , x_{i2} ,..x_{in} } \right)$$ segregation. In this process, the $$ K$$ split up is used for $$ P$$ extraction such that $$ x\left( {W_{j} } \right)$$ is valid at any one translation instance. Therefore the available components are used for $$ K$$ such that new $$ P$$ is used for achieving precise structure analysis (Table 3). By confirming the complete translation of the sentence in this initial layer, the model aims to provide accurate and comprehensive translations of the entire input sentence. The process of checking whether the full sentence is translated or not described by the Eq. (4) as followed

4 $$ L_{j}^{w} = \mathop \sum \limits_{n = 1}^{{}} \left\{ {1;\mathop \sum \limits_{k = 1}^{{}} \frac{{x_{i} w_{j} }}{W}} \right\} L_{j} = \mathop \sum \limits_{n = 1}^{{}} \left( {\frac{{\sqrt {\left( {w_{i} \left( {x_{i} } \right) - w_{j} \left( {x_{j} } \right)} \right)} }}{{\mathop \sum \nolimits_{n = 1}^{{}} \left( {P_{ij} } \right)}}} \right) L_{ij} = \mathop \sum \limits_{n = 1}^{{}} \left( {w_{i} \left( {P_{i} } \right) + w_{j} \left( {P_{j} } \right)} \right) \} $$

where $L$ is represented as the full sentence verification operation. The checking process is proceeding along with the translation output and then the input texts $\left( {L_{ij} = \mathop \sum \limits_{n = 1}^{{}} \left( {w_{i} \left( {P_{i} } \right) + w_{j} \left( {P_{j} } \right)} \right)} \right)$. Now the significance authentication process takes place in the second layer of the deep learning technique. In the deep learning process of translation, layer 2 focuses on meaning checking. After the completion of sentence translation in layer 1, layer 2 analyzes the translated output to ensure that the intended meaning of the original sentence is preserved. Examining meaning and context ensures message correctness and appropriateness. Translation requires learning source language characteristics to ensure meaning is preserved. Let can assess the translated sentence’s meaning using a deep learning method, as demonstrated in Eq. (5) below:

5 $$ P_{ij} \left( {w + x} \right) = \frac{{\mathop \smallint \nolimits_{{}}^{{}} \sigma_{x} \left( X \right)xdx}}{{\mathop \smallint \nolimits_{{}}^{{}} \sigma_{x} \left( W \right)wdw}} P_{ij} \left( {w_{i} } \right) = \frac{{\mathop \smallint \nolimits_{{}}^{{}} \sigma_{w} \left( X \right)xdx}}{{\mathop \smallint \nolimits_{{}}^{{}} \sigma_{w} \left( {x_{i} } \right)x_{i} dx_{i} }} P_{ij} \left( {x_{i} } \right) = \frac{{\mathop \smallint \nolimits_{{}}^{{}} \sigma_{x} \left( w \right)wdw}}{{\mathop \smallint \nolimits_{{}}^{{}} \sigma_{x} \left( {w_{i} } \right)w_{i} dw_{i} }} \mathop \sum \limits_{n = 1}^{{}} P_{ij} \left( {W + L} \right) = \frac{{\mathop \smallint \nolimits_{{}}^{{}} L_{ij} \left( {P + W} \right)}}{{\mathop \smallint \nolimits_{{}}^{{}} L_{ij} \left( {P_{ij} + W_{ij} } \right)}} \} $$

**Figure 2:**
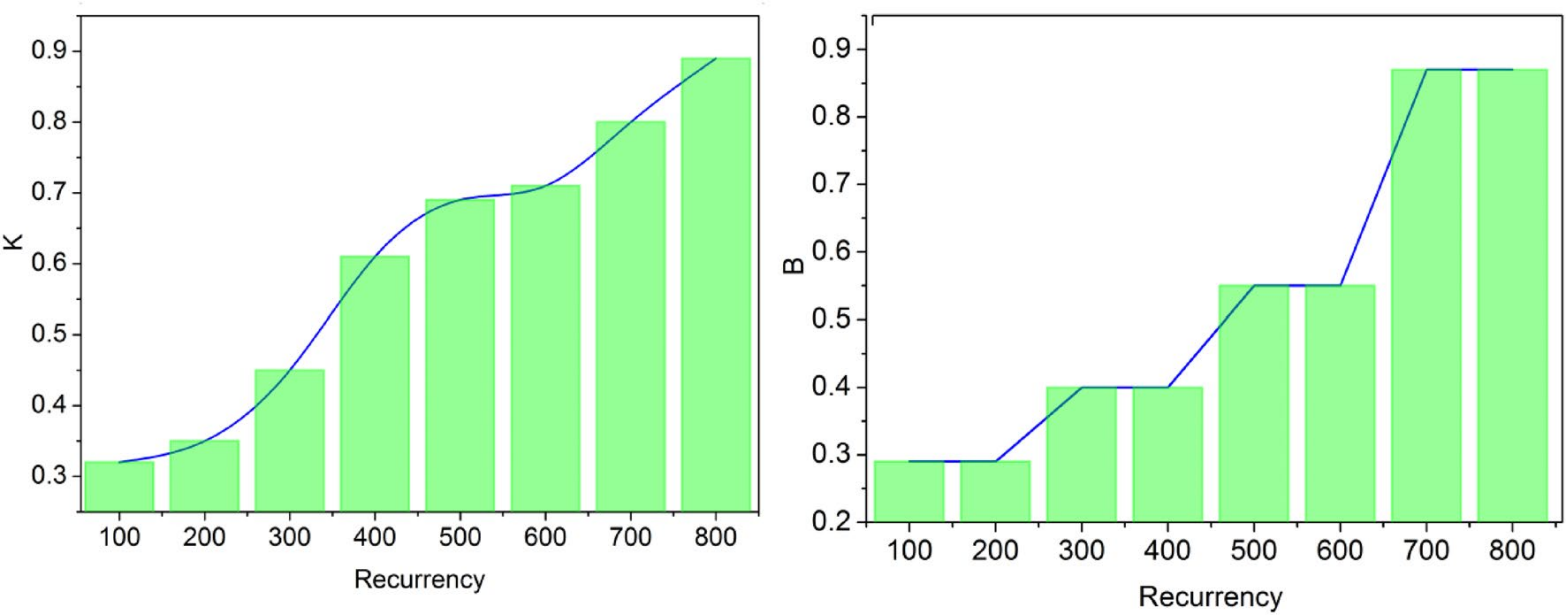
Maximum completion and meaning verification.

The study checks the content retrieval mechanism at this point. Make sure the translation matches the original after the study receives it. Concept verification using deep learning translation methods ensures that the text being translated accurately conveys the original text’s meaning. Deep learning models analyze the input phrase’s semantic representations in context to translate it. To ensure that the translated text accurately expresses the same meaning as the original, the next step is to compare the two. This verification helps to ensure that the translated text accurately captures the intended message and maintains semantic fidelity between the source and the English language. These two layers of the deep learning operation are recurrent until it detects the maximum output with precise meaning and completion. If layer-1 gets failed during the translation process, then it does not enter into the next layer-2 process. The process of verifying whether it gains the complete meaning or not is described by the Eq. (6) as followed:

6 $$ BP_{ij} = \{ 1 if b > x e^{{1 - \frac{m}{x}}} if m \le x Bw_{i} = BP.\mathop \sum \limits_{n = 1}^{{}} W_{n} P_{ij} \mathop \sum \limits_{n = 1}^{{}} \left( {w_{1} \ldots w_{n} } \right) = \mathop \sum \limits_{n = 1}^{{}} \left( {\frac{{\mathop \sum \nolimits_{i = 1}^{{}} W_{n} x_{i} }}{{\mathop \sum \nolimits_{j = 1}^{{}} W_{n} x_{j} }}} \right) = \mathop \sum \limits_{n = 1}^{{}} \left( {\frac{{W \times m\left( {P_{ij} } \right)}}{{m\left( {P_{ij} } \right)}}} \right) = \mathop \sum \limits_{n = 1}^{{}} \left\{ {b\left[ {\mathop \sum \limits_{n = 1}^{{}} \left( {\frac{{L_{ij} }}{L\left( m \right)}} \right)} \right]} \right\} \} $$

where $B$ is represented as the meaning verification process. Now the output of the deep learning process is sent to the fuzzy algorithm procedure to determine the best afford sentences after the translation process. The maximum completion and meaning verification based on the learning paradigm (recurrent steps) are analyzed in Fig. 2.

In the learning recurrency the $$ K$$ and $$ B$$ are examined in the above Fig. 2. The $$ b > x$$ and $$ m \le x$$ conditions are used at repeated $$ BP_{ji} \forall W_{n} P_{ij}$$. This is used in both layers of the learning process recurrently; this is used for all the English words that are input under multiple $$ S_{k} \left( {X,Y} \right)$$ for $$ \frac{{\left| {X \cap Y} \right|}}{\left| X \right|}$$ such that $$ X \otimes L_{y}$$ is valid for multiple $$ y \le x$$ conditions. Therefore the available $$ \left( {i \oplus_{{}}^{{}} j} \right) $$ such that $$ K$$ is the maximum. It is to be noted that for the maximum $$ K$$ the $$ B$$ is limited for $$ k = i$$ and $$ k = j$$ conditions. Therefore, the consecutive recurrent analysis is performed from the previously known best output (Fig. 2). After the two-layer process of deep learning in the translation system, determining the best-afford output is achieved using a fuzzy algorithm. The fuzzy algorithm applies linguistic rules, similarity measures, and fuzzy logic to evaluate and rank the candidate translations based on their suitability and closeness to the intended meaning. It takes into account factors such as grammar, syntax, semantics, and context to select the most appropriate translation from the available options. By leveraging the fuzzy algorithm, the translation system aims to improve the precision and accuracy of the final output, ensuring that it aligns well with the perfect meaning of the original text. The process of determining the best afford output with the benefit of the fuzzy logic algorithm is described by the subsequent Eq. (7) given below:

7 $$ S_{K} \left( {X,Y} \right) = \frac{{\left| {X \cap Y} \right|}}{\left| X \right|} S_{K} \left( {X,Y} \right) = \frac{{\mathop \sum \nolimits_{i = 1}^{n} \left( {\sigma_{X} \left( {x_{i} } \right),\sigma_{Y} \left( {x_{i} } \right)} \right)}}{{\mathop \sum \nolimits_{i = 1}^{n} \left( {\sigma_{X} \left( {x_{i} } \right)} \right)}} S_{{m_{j} }} \left( {X,Y} \right) = \frac{{\mathop \sum \nolimits_{i = 1}^{n} \left( {\sigma_{X} \left( {x_{i} } \right),\sigma_{Y} \left( {x_{i} } \right)} \right)}}{{\mathop \sum \nolimits_{j = 1}^{n} \left( {\sigma_{X} \left( {x_{i} } \right),\sigma_{Y} \left( {y_{i} } \right)} \right)}} S_{j} \otimes L_{ij} = \mathop \sum \limits_{n = 1}^{{}} \left( {x + y - L_{ij} } \right) X \otimes L_{y} = \mathop \sum \limits_{n = 1}^{{}} \left( {X,Y} \right) \left| {X,Y} \right| = \left\{ {x \in L; x < 1; y \le \underline {x} } \right\} \} $$

where $S$ is represented as the best-afford output determined by the fuzzy algorithm. The best afford output is claimed after estimating whether the translated sentence has the highest completion and most nearing meaning of the given input in other language texts. The fuzzy logic algorithm is utilized to estimate whether the translated sentence achieves the highest completion and closest meaning to the given input text in another language. The best-afford solution instances from the second learning layer filtered are analyzed in Table 4.

**Table 4:** Best-afford solution instances.

$W$**(K)**	$L$	$x < 1$	$y \le \underline {x}$	$A \otimes L_{i}$	$A \otimes W_{i}$	Best-Afford Instances
1	0.3	●	●	●	●	**●**
	0.6	●	●	●	●	**●**
	0.9	●	●	●	●	**●●**
2	0.3	●	●	●	●	**●**
	0.6	●	●	●	●	**●**
	0.9	●	●	●	●	**●●**
3	0.3	●	●	●	●	**●**
	0.6	●	●	●	●	**●●**
	0.9	●	●	●	●	**●**
4	0.3	●	●	●	●	**●**
	0.6	●	●	●	●	**●**
	0.9	●	●	●	●	**●**
5	0.3	●	●	●	●	**●**
	0.6	●	●	●	●	**●**
	0.9	●	●	●	●	**●**

The best-afford instance from two different conditions $$ \left( {x,y} \right)$$ and $$ \left( {A,L} \right)$$ are analyzed in Table 4. This is required for handling two conditions of $$ x < 1$$ and $$ y \le \underline {x}$$ wherein the inputs determine the precise extractions. The best-afford solutions are valid if $$ A \otimes L$$ and $$ A \otimes W$$ are valid for $$ P(\left( {X,Y} \right)$$. Depending on $$ \underline {K}$$ and $$ \sigma \underline {K} \left( x \right)$$, $$ \sigma \left( x \right)$$ is used for analyzing the $$ i \forall K$$. If the changes are valid for multiple instances then $$ B$$ is utilized for $$ x_{j}$$ such that $$ \sigma \otimes W_{i}$$ is true. In this case, the best-afford solution is achieved if $$ \left( {A \otimes L_{i} } \right)$$ and is true. Therefore the $$ W\left( K \right)$$ is available for better improvement (Table 5). By considering linguistic rules and similarity measures, the algorithm determines the best afford output by evaluating the grammatical accuracy, syntactic coherence, and semantic proximity of the translation, ensuring a high-quality and contextually appropriate result. The process of verifying the translated sentence has the highest completion and nearest meaning is described by the Eq. (8) as followed

8 $$ a_{kl} = \{ a_{ij} for k = i, k = j a_{kj} otherwise x_{k} = \left\{ {x_{i} for k = 1 x_{j} otherwise x_{j} = \left( {i \oplus j,n_{ij} } \right) \le \left( {i \oplus j,L_{j} } \right) = \left( {i \oplus j,L_{ij} } \right) + \left( {i \oplus j,W_{ij} } \right) A \otimes L_{i} = \sigma \otimes L_{i} A \otimes W_{i} = \sigma \otimes W_{i} } \right\} $$

where $A$ is represented as the proximity of the translation. Now the errors are detected from the output to clarify it by using the fuzzy algorithm. The detection of errors in the best afford outputs is facilitated by the fuzzy algorithm. The algorithm compares the translated output with the original input and identifies discrepancies or errors by evaluating the linguistic rules, contextual coherence, and semantic similarity. It applies fuzzy logic to assess the degree of deviation from the desired translation quality. By detecting and highlighting errors, the fuzzy algorithm helps expand the accuracy and precision of the changes, enabling refinement and ensuring a more reliable output which is defined in Eq. (9 and 10).

9 $$ X \cup Y = \left\{ {\left( {x \in A} \right) \cup \left( {x \in B} \right)} \right\} X_{A \cup B} \left( x \right) = \mathop \sum \limits_{n = 1}^{{}} \left( {\sigma_{A} \left( x \right), \sigma_{B} \left( x \right)} \right) \sigma_{A} \left( x \right) = 1 \sigma_{A} \left( x \right) = \sigma_{B} \left( x \right) = 0 X \cap Y = \left\{ {\left( {x \in A} \right) \cap \left( {x \in B} \right)} \right\} X_{A \cap B} \left( x \right) = \mathop \sum \limits_{n = 1}^{{}} \left( {\sigma_{A} \left( x \right), \sigma_{B} \left( x \right)} \right) \sigma_{A} \left( x \right) = 1 \sigma_{A} \left( x \right) = \sigma_{B} \left( x \right) = 1\} $$

10 $$ \underline {X} = \left\{ {x \in A} \right\} \sigma_{{\underline {X} }} \left( x \right) = 1 - \sigma_{A} \left( x \right) \sigma_{A} \left( x \right) = 0 \underline {K} = \left\{ {k \in B} \right\} \sigma_{{\underline {K} }} \left( x \right) = 1 - \sigma_{K} \left( x \right) \sigma_{K} \left( x \right) = 1 \sigma \left( x \right) = \sigma \left( {x_{i} } \right)\} $$

**Table 5:** $$ \sigma$$ identified.

$A$	$j$	$B$	$X \cup Y$	$X \cap Y$	$\sigma$
0	0	0	0	0	0.19
0	0	1	0	0	0.27
0	1	1	1	0	0.09
0	1	0	0	0	0.21
1	0	0	0	0	0.15
1	0	1	0	0	0.12
1	1	0	0	1	0.058
1	1	1	1	1	0.03

The process of estimating the errors from the best affordable solutions is explained by the following equations given above. Where $$\sigma$$ is represented as the detected errors in the best afford outputs. Now the sequences are determined after detecting the errors by using the fuzzy logic algorithm. The fuzzy logic algorithm is utilized to detect the sequence of the best affords output in the translation process. In Table 5 the $$ \sigma$$ identified from the $$ j$$ and $$ A$$ is analyzed.

Based on $$ A,j,$$ and $$ B$$ the $$ \left( {X \cup Y} \right)$$ and $$ \left( {X \cap Y} \right)$$ are analyzed for varying inputs. In the fuzzification process, the best-afford solution is obtained based on $$ \sigma_{B} \left( x \right) = 1$$. The best-afford filtration using $$ \left( {A,W} \right)$$ and $$ \left( {A,L} \right)$$ is congruent for improving proximity. Hence the learning process is recurrent access multiple $$ P$$ such that $$ x_{j} = \left( {i \oplus j} \right) \le \left( {i \oplus j} \right)\forall L_{j}$$. This is optimal for increasing the completion with precise meaning improvement. Therefore, the errors are detected from the previous state of the learning process for maximizing translation accuracy (Table 5). By considering linguistic rules, similarity measures, and fuzzy logic principles, the algorithm analyzes the translated output and determines the optimal sequence of words and phrases. The development of determining the sequences of the output by using the fuzzy logic described by the Eq. (11) as followed

11 $$ \left( {\underline{S + r} } \right)\left( Z \right) = \underline {S} \left( Z \right) + r\left( Z \right) \left( {\underline{S + r} } \right)\left( Z \right) = \underline {S} \left( Z \right) + \underline {r} \left( Z \right) K\underline {r} = \{ \left( {K\underline {P} \left( Z \right),K\underline {r} \left( Z \right)} \right), K \ge 0 \left( {K\underline {r} \left( Z \right),K\underline {P} \left( Z \right)} \right), k < 0 \tilde{S} = \tilde{r} = \underline {S} \left( Z \right) = \underline {r} \left( Z \right) = 0 \underline {S} \left( Z \right) = W - \left( {1 - Z} \right)\sigma \underline {S} \left( r \right) = W + \left( {1 - Z} \right)P \underline {S} - \underline {S} = \left( {\sigma + P} \right)\left( {1 - Z} \right)\} $$

Where $r$ is represented as the sequence of the obtained texts, $Z$ is represented as the output of the error determination process defined in Eq. (12).

12 $$ G = \left( {w s s w } \right) \{ G_{11} \left( Z \right) + \underline {S} \left( r \right) + \ldots + G_{1n} \left( Z \right) + \underline {S} \left( r \right) = \underline {w}_{1} \left( Z \right) + \underline {w}_{1} \left( Z \right) G_{21} \left( Z \right) + \underline {S} \left( r \right) + \ldots + G_{2n} \left( Z \right) + \underline {S} \left( r \right) = \underline {w}_{2} \left( Z \right) + \underline {w}_{2} \left( Z \right) G_{n1} \left( Z \right) + \underline {S} \left( r \right) + \ldots + G_{nn} \left( Z \right) + \underline {S} \left( r \right) = \underline {w}_{n} \left( Z \right) + \underline {w}_{n} \left( Z \right) \underline {S} \left( Z \right) = (w + S)^{ - 1} \left( {\underline {w} \left( Z \right)} \right) \underline {S} \left( r \right) = (w + S)^{ - 1} \left( {\underline {w} \left( Z \right)} \right) G^{ - 1} = \left( { - w s s - w } \right) \} $$

The best afford output is detected by contemplating the errors and then the translation procedure $\left( {\underline {S} - \underline {S} = \left( {\sigma + P} \right)\left( {1 - Z} \right)} \right)$. In this process, it verifies where the best afford output is located whether in layer-1 or layer-2. The process of checking the location of the acquired best afford output is described by the Eq. (12) as followed:

Where $G$ is denoted as the location of the best afford output. Now the modification is preceded by the deep learning process to establish the enhanced translated output without any errors. The translated result is decided error-free after using the fuzzy logic procedure. The improved translated output raises the bar for translation quality by accurately conveying the source text’s meaning in the target language. The process of obtaining the translated output after the fuzzy algorithm process is described by the Eq. (13 and 14) as followed:

13 $$ \{ \underline {U} \left( Z \right) = \frac{G\left( Z \right) - w\left( Z \right)}{2} \underline {U} \left( Z \right) = \frac{G\left( Z \right) + w\left( Z \right)}{2} \{ \underline {U} \left( Z \right) = \frac{{G\left( Z \right) - w\left( {1 - Z} \right)}}{2} \underline {U} \left( Z \right) = \frac{{G\left( Z \right) + w\left( {1 - Z} \right)}}{2} \{ S_{n} - w_{n} = 2n^{2} S_{n} - w_{n} = Z_{n} \left( x \right) + n^{2} \{ K_{n} - L_{n} = n^{2} K_{n} - L_{n} = W_{n} \left( Y \right) + n^{2} \left\{ {Bw + Aw = n^{2} \frac{{\mathop \sum \nolimits_{n = 1}^{{}} Aw}}{2n} = \mathop \sum \limits_{n = 1}^{{}} \left( {x_{ij} } \right) } \right\} $$

14 $$ \varphi = 1 - \frac{1}{{\frac{s}{r} + \frac{1 - s}{r}}} \varphi_{b} = 1 - s \varphi = \frac{1}{{1 + w^{2} }} \varphi_{x} = \{ 1 if x \in A 0 if x \notin A \varphi_{sj} = \{ 0 if S \le 0 1 if S > 0 \} $$

$$ U$$ stands for the output of the fuzzy algorithm, whereas $$ \varphi$$ represents the translated output. Using a combination of a fuzzy logic method and a deep learning approach during translation yields improved translated output. Improved completion accuracy and, by extension, output meaning, are both aided by this procedure. Not only that, it improves word flexibility while decreasing mistake rates. Research into languages and translations must concentrate on the flexibility of words in order to guarantee that translated texts adequately express nuance, context, and fluency. Not only does it improve user experiences with translated information, but it also preserves original purpose and increases cross-cultural awareness. Enhanced translation quality, more user trust, time and money saved, and a competitive edge in machine translation and language services are some of the expected consequences. Word adaptability is an important part of language technology and fluency because it helps people communicate more clearly and engage more successfully across languages. Table 6 presents the validation output of word adaptability/ sentence analyzed.

**Table 6:** Validation output of word adaptability/sentence.

	$X \cup Y$	$X \cap Y$	$U$	$r$ Per Word	$A \otimes L_{i}$	$A \otimes W_{i}$	$G$	$\varphi$
$x < 1$	0	0	0.5	18	●●	●●	14	**0**
	0	1	0.58	17	●●	●●	15	**1**
	1	0	0.71	12	●●	●●	13	**0**
	1	1	0.85	13	●●	●●	12	**0**
$y \le \underline {x}$	0	0	0.6	15	●●	●●	14	**1**
	0	1	0.72	13	●●	●●	15	**1**
	1	0	0.81	12	●●	●●	14	**0**
	1	1	0.93	13	●●	●●	11	**1**

In Table 6, the $$G$$ and $$ \psi$$ analysis for two outputs $$ X \cup Y$$ and $$ X \cap Y$$ is analyzed. Based on the available $$r \forall \left( {x < 1} \right)$$ and $$ (y \le \underline {x}$$) the $$ \left( {A \otimes L} \right) $$ and $$ A \otimes W$$ are congruently allocated for better improvement. The fuzzy output is used for improving the training rate regardless of the $$ X \cup Y$$ Output. Therefore utilizing the $$ \psi \forall G$$ and $$ \left( {X \cap Y} \right)$$ validates the identifying unit across various $$ i$$. Thus the maximum $$ G$$ is confined between $$ r$$ (per wordy sentence), for which new improvements are performed. Therefore, the recurrence is utilized for providing optimal outputs across various inputs (words/sentences). Then the step by step process explained as follows.

Algorithm Steps for FPTA:

- Step 1: Load the translation input to the system.
- Step 2: Convert the text into the English language according to Eq. (2)
- Step 3: Extract the semantics (words) from the sentence.
- Step 4: Checking the completion of the translated sentence by using the deep learning technique using Eq. (3)
- Step 5: Analyze the sentence words, components, and structure to perform the translation process using layers of networks.
- Step 6: aligns the text with improving the overall translation accuracy using fuzzy rules.
- Step 7: The process of determining the best-afforded output with the help of the fuzzy logic algorithm is explained using Eq. (7)
- Step 8: According to the fuzzy rules, the translation sequences are decided to improve the translation accuracy.

## Performance assessment

Measures including word utilization, versatility, understandability, verification time, and mistakes are used to evaluate performance. Sentences are evaluated up to 7000 words long and employ 12-word placements. The suggested technique for performance evaluation is enhanced using the techniques NMT-LFE^29^, SLSE-DNN^24^, and MNMT^22^. To measure the efficacy and precision of translations, an exclusive set of metrics is used for the assessment. Some of these metrics include “Word Adaptability,” a measure of the extent to which each method adapts words from one language to another; “Understandability,” which measures the likelihood that a native speaker of the target language understands the translations; “Verification Time,” which measures the time needed to check the translations for accuracy and quality; “Errors,” which quantifies the amount and kinds of mistakes, including grammatical, contextualized, and factual inaccurate information; and “Word Usage,” which judges how well the translated sentences make use of appropriate vocabulary. Every sentence must have at least twelve words and no more than seven thousand characters in length to be considered substantive. The assessment approach begins by translating the chosen sentences using all of the ways. Then, every methodology is valued according to the metrics that are defined. Statistical methods may be used to determine the degree of performance difference between the approaches. With the results presented in such an organized and comprehensive fashion, the benefits and drawbacks of each approach may be better understood, and practical applications can be proposed. Let’s construct the framework in MATLAB and then validate it using the metrics to evaluate the way it works.

### Results analysis

#### Word adaptability

**Figure 3:**
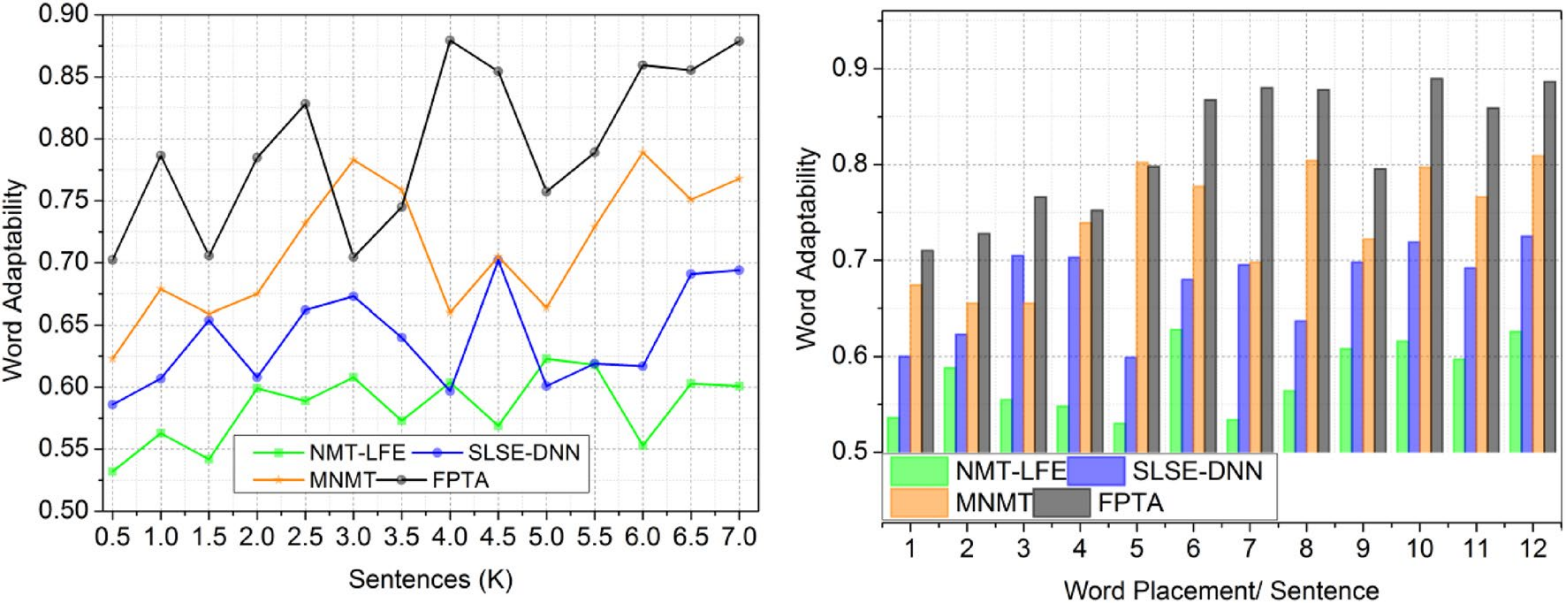
Word adaptability.

To ensure complete and relevant material, deep learning is used. Highly adaptable English-to-Spanish translation requires deep learning. Deep learning improves cross-lingual translation, allowing effective English translations from complex source materials. Better translators can be trained by feeding deep learning models additional data and modifying their output. Because flexibility produces more accurate as well as natural translations, translation becomes more efficient. With this data, algorithms may detect patterns in phrases, idioms, and more precise keywords in mountains of data. Deep learning and the fuzzy algorithm are taught and refined to translate languages, as seen in Fig. 3. It also enhances “adaptability” perceptions.

#### Understandability

**Figure 4:**
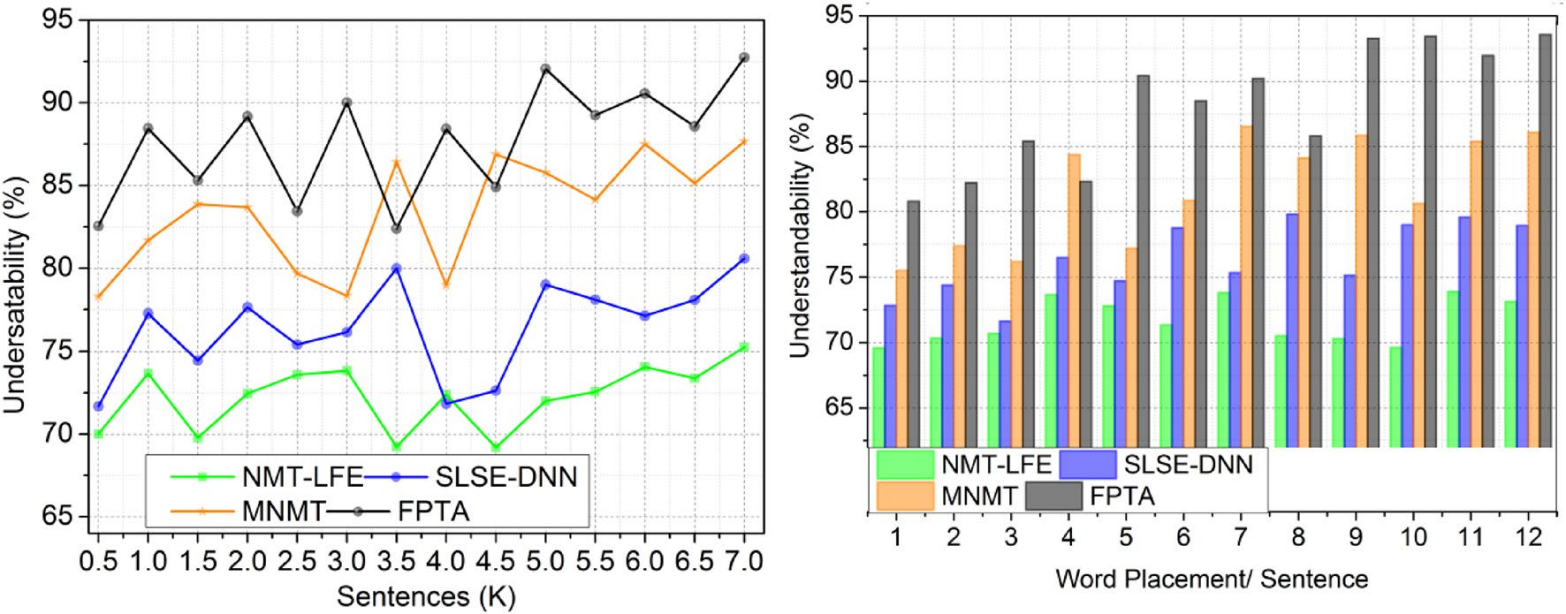
Understandability.

The study found that fuzzy algorithms could make this procedure easier and more accurate. Deep learning and fuzzy logic may improve English-to-Spanish translations. Fuzzy logic takes into account linguistic confusion and unpredictability to create a more complex source language image. Understanding linguistic correlations and patterns requires deep learning models to learn from large datasets. Including fuzzy logic in deep learning model training improves translation accuracy while keeping context. Integration improves translation accuracy and clarity, enabling cross-lingual communication and reducing translation ambiguities. After considering all attributes and procedures, Fig. 4‘s recommended method its visual representation is easier to understand.

#### Verification time

**Figure 5:**
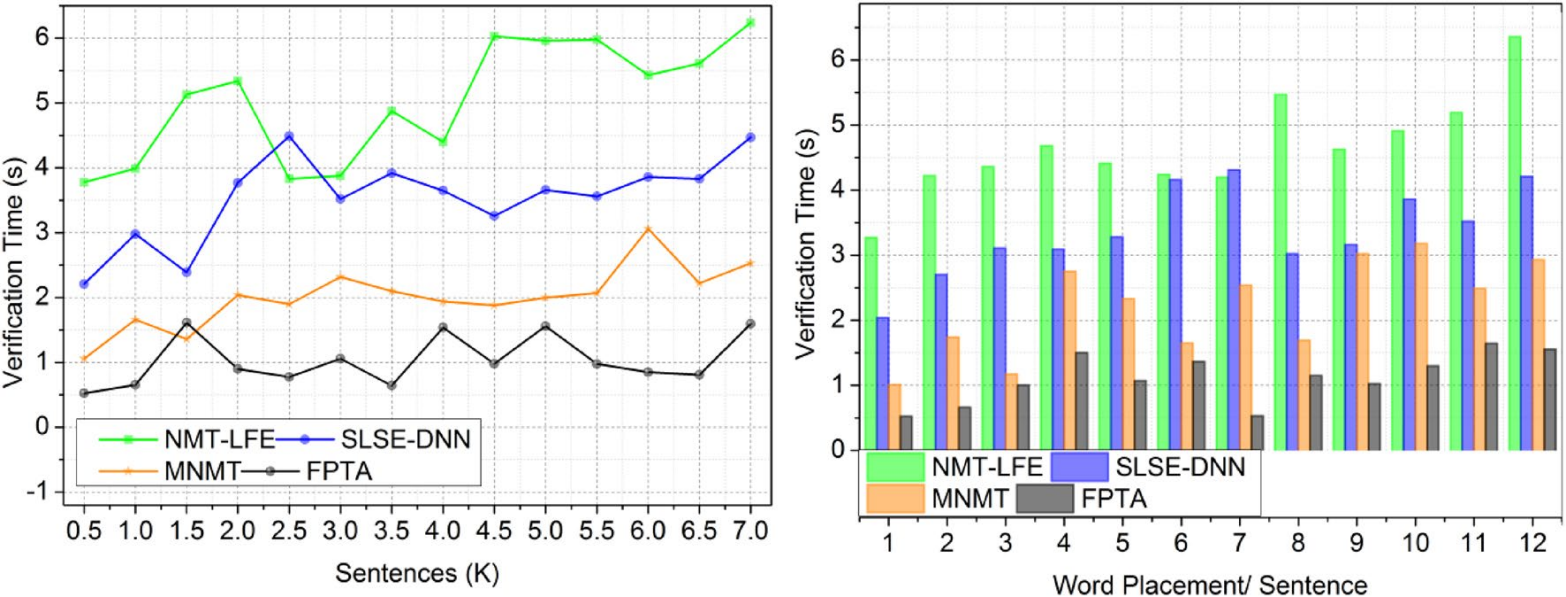
Verification time.

Deep learning results reduce verification time in this approach. Two deep learning levels speed up translated output accuracy checks. Deep learning trains translation to identify connections and trends in source and target languages using a vast amount of matched bilingual data. Through this first-layer approach, precise translations are made possible. Another layer, meaning verification, helps the translation model understand the input sentence. Other deep learning approaches take longer to implement than the two-layer method. A more efficient method that allows instantaneous translation output review speeds up the process. The research advises using deep learning to speed up verification. See Fig. 5 for confirmation time visualization.

#### Errors

**Figure 6:**
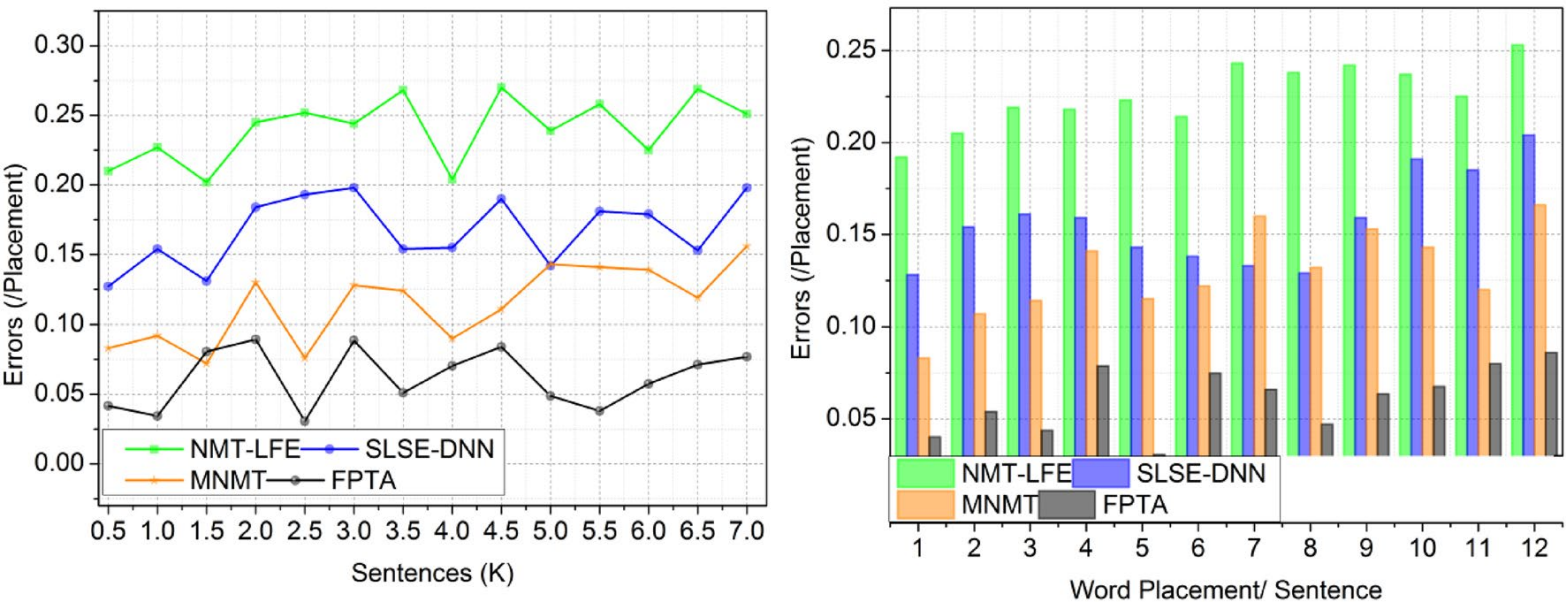
Errors.

To minimize the amount of errors in the process, the fuzzy technique is used after deep learning has detected the completeness overall meaning of the translation. In order to drastically lower the rate of errors in English translation, fuzzy algorithms can be used. Incorporating linguistic ambiguity and imprecision, fuzzy algorithms expand the capabilities of language interpretation. Contextual accuracy is enhanced by translations that account for the various meanings of words and phrases depending on their context. In their language analysis, fuzzy algorithms consider word relationships, synonyms, and ambiguity. Complex phrase patterns and informal idioms are now handled significantly more adeptly by the translation process due to this update. Figure 6 shows how fuzzy algorithms are used to improve the quality and accuracy of translated output by reducing the impact of precise translations and misinterpretations.

#### Word usage

**Figure 7:**
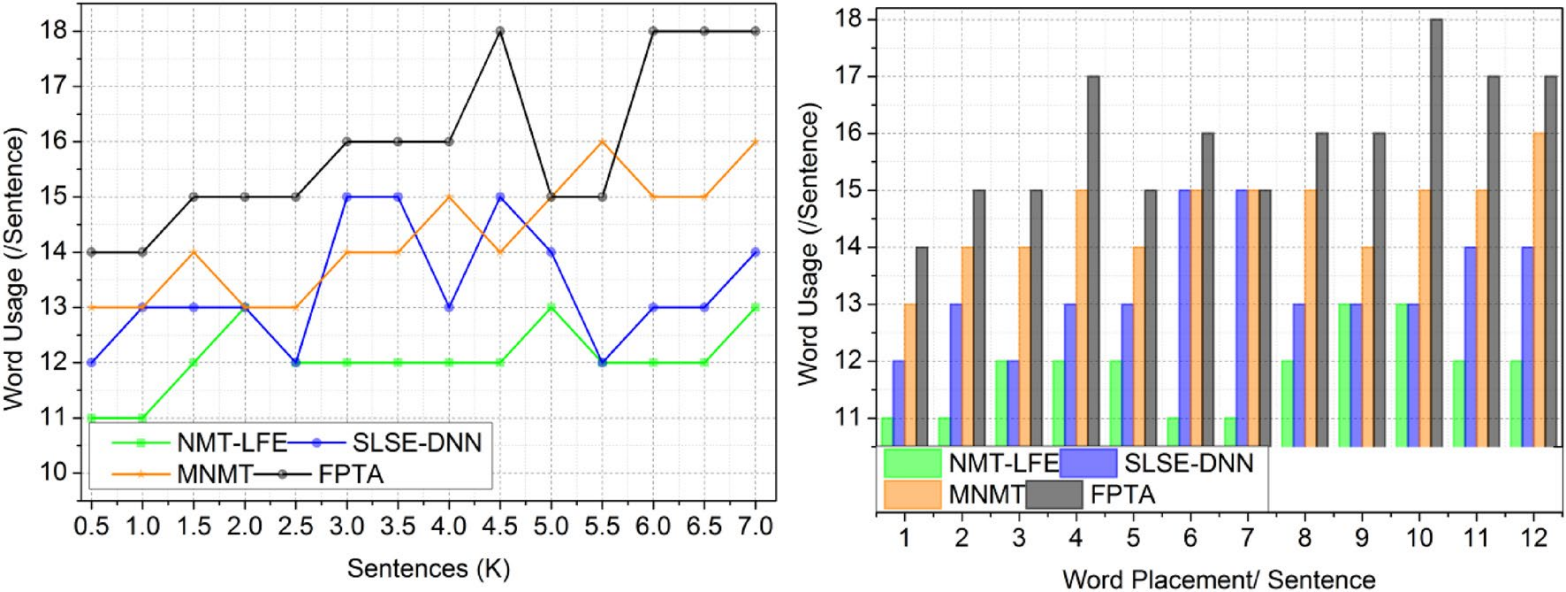
Word usage.

Due to its incorporation of fuzzy evaluation and deep learning findings, its application in this approach yields desirable outcomes. It is possible that translation systems might provide better, more accurate, and context-appropriate translations of words and phrases by utilizing deep learning techniques. Any English translation procedure worth its salt will include a final check for correctness and meaning to ensure the usage of effective terminology. Because of the importance of accurately conveying meaning in translated information, this validation stage is crucial. Making sure the translated phrases are grammatically and syntactically correct is what this process is all about. Another component is checking that the chosen words and phrases are true to the original content’s intended meaning. The translation process strives for linguistic correctness and the effective and clear conveyance of the original message in the target language by checking the final result and meaning of the converted output (Fig. 7).

Table 6 summarizes the comparative analysis results.

## Discussions

In this part, the study takes a look at the outcomes that FPTA achieved while translating from English to English remotely. Current methods like NMT-LFE, SLSE-DNN, and MNMT are used to compare the system’s efficiency. The findings are presented in Table 7.

**Table 7:** Comparative analysis results.

Metrics	NMT-LFE	SLSE-DNN	MNMT	FPTA	Findings
Sentences					
Word Adaptability	0.601	0.694	0.768	0.8788	9.56% High
Understandability (%)	75.26	80.59	87.67	92.741	11.57% High
Verification Time (s)	6.24	4.47	2.53	1.594	10.66% Less
Errors (Placement)	0.251	0.156	0.198	0.0768	12.49% Less
Word Usage (/Sentence)	13	14	16	18	10.19% High
Word Placement/ Sentence					
Word Adaptability	0.626	0.725	0.809	0.8864	8.32% High
Understandability (%)	73.1	78.93	86.09	93.563	14.2% High
Verification Time (s)	6.36	4.21	2.93	1.555	10.91% Less
Errors (Placement)	0.253	0.204	0.166	0.086	12.17% Less
Word Usage (/Sentence)	12	14	16	17	8.82% High

FPTA improves word flexibility using deep learning approaches, which results in more accurate translations. Fuzzy logic is used to improve the understandability of translated English by addressing ambiguity and vagueness included in the original language. One layer of machine learning expedites the process of ensuring translation accuracy by training the model with a large multilingual corpus. Reduced error rates caused by linguistic imprecision and uncertainty is one way fuzzy computers improve the output quality and accuracy of translations. The core of FPTA is a combination of deep learning and fuzzy assessment that improves linguistic precision in the target language, addressing accuracy difficulties in remote translationUtilize FPTA, a translation technology that integrates deep learning with fuzzy logic, for more accurate translations and simpler human comprehension.Due to its ability to handle a wide range of sentence patterns, idioms, and terminology, it renders translations more naturally. Training using Fuzzy Logic enhances clarity and readability by factoring in linguistic ambiguity. In order to verify translations more quickly and efficiently, FPTA employs a two-tiered deep learning strategy. In order to decrease the number of errors, fuzzy algorithms consider uncertainty and ambiguity in language. Results are both semantically and linguistically exact when deep learning is combined with fuzzy assessment.

## Conclusion

In this article, the fusion-dependent precision translation approach is designed and discussed for improving the understandability of remote English translations. The precision problem due to on-demand and low-resource translations is addressed using two different learning layers recurrently. The maximum affordable solution for understandability and word usage for sentence completion is validated for improving the precision. In the fuzzy process, the learning model’s training is determined using the best-afford solution. Besides, the word placement and its precision for completion are classified by the fuzzy process for converging the training. In the training process, the solutions are tuned towards the maximum affordable solution that reduces the errors. Therefore the error sequence identification from the first to the final layer of the learning process is persistent such that the errors are reduced. From the data-based analysis, it is seen that the proposed approach improves understandability by 11.57% and reduces error by 12.49% for different translated sentences. However, the proposed approach operates within certain constraints. These constraints include the availability and quality of data, as the system’s effectiveness relies heavily on these factors. Scalability concerns may arise due to resource-intensive requirements, and ensuring the interpretability of the system’s decisions is vital. Finally, the system may require time to adapt to individual students, with initial recommendations potentially lacking precision. In sum, the Fuzzy Control System for music instruction offers a structured method to tackle educational challenges but must navigate these constraints to deliver effective and tailored music instruction.

Future work in the domain of Fuzzy Control System for music instruction should strive to combine technological advancements with educational and interdisciplinary insights. The system may improve its ability to serve music students, teachers, and fans by tackling the highlighted limitations and investigating these avenues, leading to an enhanced educational experience.

### Ethical statement

Prior to their recruitment in this research, all individuals were asked to provide written informed permission.

## Data Availability

The statistics that back up the study’s conclusions are accessible in the journal, according to the author.
